# Evaluation of the TLR3 involvement during *Schistosoma japonicum*-induced pathology

**DOI:** 10.1186/s12865-023-00586-9

**Published:** 2024-01-03

**Authors:** Hongyan Xie, Dianhui Chen, Yuanfa Feng, Feng Mo, Lin Liu, Junmin Xing, Wei Xiao, Yumei Gong, Shanni Tang, Zhengrong Tan, Guikuan Liang, Shan Zhao, Weiguo Yin, Jun Huang

**Affiliations:** 1 Department of Laboratory Medicine, The Sixth Affiliated Hospital of Guangzhou Medical University, Qingyuan People’s Hospital, Qingyuan, 511518 China; 2 China Sino-French Hoffmann Institute, Department of basic Medical Science, Guangzhou Medical University, Guangzhou, 511436 China; 3 Department of Infectious Diseases, The Third Affiliated Hospital of Guangzhou Medical University, Guangzhou, 510150 China; 4 State Key Laboratory of Respiratory Disease, National Clinical Research Center for Respiratory Disease, Guangzhou, China

**Keywords:** *Schistosoma japonicum*, Liver, TLR3, Amelioration

## Abstract

**Background:**

Despite the functions of TLRs in the parasitic infections have been extensively reported, few studies have addressed the role of TLR3 in the immune response to *Schistosoma japonicum* infections. The aim of this study was to investigate the properties of TLR3 in the liver of C57BL/6 mice infected by *S. japonicum.*

**Methods:**

The production of TLR3^+^ cells in CD4^+^T cells (CD4^+^CD3^+^), CD8^+^T cells (CD8^+^CD3^+^), γδT cells (γδTCR^+^CD3^+^), NKT cells (NK1.1^+^CD3^+^), B cells (CD19^+^CD3^−^), NK (NK1.1^−^CD3^+^) cells, MDSC (CD11b^+^Gr1^+^), macrophages (CD11b^+^F4/80^+^), DCs (CD11c^+^CD11b^+^) and neutrophils (CD11b^+^ Ly6g^+^) were assessed by flow cytometry. Sections of the liver were examined by haematoxylin and eosin staining in order to measure the area of granulomas. Hematological parameters including white blood cell (WBC), red blood cell (RBC), platelet (PLT) and hemoglobin (HGB) were analyzed. The levels of ALT and AST in the serum were measured using biochemical kits. The relative titers of anti-SEA IgG and anti-SEA IgM in the serum were measured by enzyme-linked immunosorbent assay (ELISA). CD25, CD69, CD314 and CD94 molecules were detected by flow cytometry.

**Results:**

Flow cytometry results showed that the expression of TLR3 increased significantly after *S. japonicum* infection (*P* < 0.05). Hepatic myeloid and lymphoid cells could express TLR3, and the percentages of TLR3-expressing MDSC, macrophages and neutrophils were increased after infection. Knocking out TLR3 ameliorated the damage and decreased infiltration of inflammatory cells in infected C57BL/6 mouse livers.,The number of WBC was significantly reduced in TLR3 KO-infected mice compared to WT-infected mice (*P* < 0.01), but the levels of RBC, platelet and HGB were significantly increased in KO infected mice. Moreover, the relative titers of anti-SEA IgG and anti-SEA IgM in the serum of infected KO mice were statistically decreased compared with the infected WT mice. We also compared the activation-associated molecules expression between *S.japonicum*-infected WT and TLR3 KO mice.

**Conclusions:**

Taken together, our data indicated that TLR3 played potential roles in the context of *S. japonicum* infection and it may accelerate the progression of *S. japonicum*-associated liver pathology.

## Introduction

*Schistosoma japonicum*, a major infectious agent of schistosomiasis, causes morbidity and mortality, especially in developing countries [1, 2]. The intestines and liver are the sites of highest egg entrapment [3]. In addition, invasive parasite stages elicit an inflammatory reaction in skin and lungs, in which macrophage and eosinophil granulocytes are predominant [4]. The granulomatous inflammation against parasite eggs, and the subsequent fibrosis, are the pathological hallmark of Schistosoma infection. The granuloma consists of recruited lymphoid and myeloid cells [5] and is the product of complex cellular interactions with the participation of adhesion molecules and cytokines [6].

A plethora of immune cells reside in the liver, including T cells, natural killer (NK) cells, natural killer T (NKT) cells, gamma delta T cells (γδ T cells), myeloid-derived suppressor cells (MDSCs), macrophages, and others [7, 8]. They have been shown to regulate inflammatory and T cell-mediated immune responses [9]. Liver, an important organ in the regulation of peripheral immunological responses, is characterised by a remarkable ability to induce tolerance to antigens [10, 11]. *S. japonicum eggs become lodged in the liver and intestinal wall, where they induce a local granulomatous inflammation* [12]. Granulomas isolate or neutralize egg antigens but also lead to fibrogenesis in host tissues [7], which is related to chronic schistosomiasis.

Toll-like receptors (TLRs), a class of pattern recognition receptors, are able to recognize distinct pathogen-associated molecular patterns (PAMPs) and play a critical role in innate immune responses [13]. Evidence is accumulating that TLRs recognition of their specific ligands induces a signaling cascade resulting in the induction of pro-inflammatory cytokines, including type I interferon (IFN), which could drive an inflammatory response and activate the adaptive immune systems [14]. TLR engagement triggers signaling cascades involving intracellular adaptors, such as MyD88 [15]. However, it showed that the signaling pathways associated with each TLR are not identical and may, therefore, result in different biological responses [14].

TLR3 is a trans-membrane receptor that is found on intracellular organelles, which senses exogenous as well as endogenous double-stranded RNA in endosomes [16]. Several mechanisms could account for the accumulation of TLR3-activitory dsRNA molecules within endosomes, including uptake of apoptotic bodies derived from infected cells [17], clathrin-dependent endocytosis [18], autophagic uptake of dsRNA from the cytosol and trafficking to endosomes in the context of inhibited lysosomal degradation [19, 20]. TLR3 is implicated in the pathogenesis of many inflammatory diseases, such as the coronavirus disease (COVID-19) [21], herpes simplex encephalitis (HSE) [22] and *Paracoccidioides brasiliensis* infections [23]. It has been identified TLR3 as central sensor of parasite and egg components during *S. mansoni* infection [24–26]. Our previous results suggested that TLR3 might be involved in regulating the immune response of NK cells in the course of *S. japonicum* infection in the spleen [27]. The role of TLR3 in livers of mice infected with *S. japonicum*, however, is still discussed controversially.

## Materials and methods

### Mice

Six to eight-week old C57BL/6 mice were purchased from Traditional Chinese Medicine University of Guangzhou Animal Center (Guangzhou, China). All animal experiments were performed in strict accordance with the Regulations for the Administration of Affairs Concerning Experimental Animals (1988.11.1). Mice were housed at the Animal Center of Guangzhou Medical University, on a 12 hr./12 hr. light/dark cycle, with free access to food and water. The animal protocols were approved by the Committee on the Ethics of Animal Experiments of Guangzhou Medical University.

### Infection

Oncomelania hupensis snails were purchased from Chinese Institute of Parasitic Disease (Shanghai, China). The mice were infected as previous reported [11]. In short, the slide with 40 ± 5 *S. japonicum* cercariae was covered on the abdominal skin of mice in close contact for 10 min. Then 30 mice were euthanized 6 weeks after infection. Pathogen-free C57BL/6 mice were used as controls. At 1, 2, 3, 4, 5, 6 and 7 weeks post-infection, three mice were randomly chosen and sacrificed by cervical dislocation. All animals were anesthetized with isoflurane using closed chambers (The concentration is 3% ~ 4%, the output gas flow rate is 300 ~ 500 ml/min and the time is 2 ~ 3 minutes) until after coma and sacrificed by cervical dislocation. Animals are monitored daily, and 20% weight loss is considered a humane endpoint. In this study, no cases of animal deaths were found. The animal protocols were approved to be appropriate and humane by the institutional animal care and use committee of Guangzhou Medical University. We do our best to minimize animal suffering.

### Antibodies

APC-conjugated anti-mouse TLR3 (11F8), APC-cy7-conjugated anti-mouse CD3 (145-2C11), FITC-conjugated anti-mouse CD3 (17A2), PerCP-cy5.5-conjugated anti-mouse CD4 (RM4–5), PE-conjugated anti-mouse CD8 (53–6.7), BV510-conjugated anti-mouse γδ TCR (GL3), PE-cy5-conjugated anti-mouse CD19 (6D5), PE-cy7-conjugated anti-mouse NK1.1 (PK136), APC-cy7-conjugated anti-mouse CD11b (M1/70), PE-conjugated anti-mouse Ly6G (1A8), PE-cy7-conjugated anti-mouse F4/80 (EMR1, Ly71), PE-conjugated anti-mouse Gr1(RB6-8C5), APC-conjugated anti-mouse CD69 (H1.2F3), PE conjugated anti-mouse Ly6G (1A8-Ly6g), PerCP-Cy5.5-conjugated anti-mouse CD11c (HL3), PE-anti-mouse CD25 (BC96), APC-anti-mouse CD69 (H1.2F3), FITC-conjugated anti-mouse CD94 (20d5), PE-conjugated anti-mouse CD314 (XMG1.2) were purchased from BioLegend (San Diego, CA, USA) and BD Pharmingen (San Diego, CA, USA).

### Non-parenchymal liver cells isolation

Mice were perfused with sterile saline to remove blood from the body before obtaining the liver tissue. Analysis of livers cells was performed on cell suspensions obtained from organ homogenates that were digested with a LIVER dissociation kit (Miltenyi Biotec, Germany). Non-parenchymal liver cells were isolated by Ficoll-Hypaque (DAKEWE, SZ, China) density gradient centrifugation from the cell solution [11]. They were washed in HBSS and resuspended at 2 × 10^6^ cells/ml in complete RPMI 1640 medium. The number of cells was measured under a microscope (Olympus, Shinjuku, Tokyo, Japan).

### Flow cytometry detection

Cells were then stained with conjugated antibodies specific for the cell surface antigens for 30 min. Then, expressions phenotypes of antibody-labeled cells were analyzed by flow cytometry (Beckman CytoFLEX), and the results were analyzed using the CytExpert 1.1 (Beckman Coulter Inc.). Isotype-matched controls for cell surface markers were included in each staining protocol. When the samples were stained for TLR3, cells were fixed with fixation and permeabilization solution (BD Biosciences) for 20 min at 4 °C in the dark. Then, cells were permeabilized by PBS buffer containing 0.1% saponin (Sigma), 1% BSA, and 0.05% NaN_3_ overnight at 4 °C. Next, cells were stained with conjugated antibodies that were specific for TLR3.

### Histology studies

Livers were removed from mice, perfused with 0.01 M phosphate-buffered saline (pH = 7.4) for three times, fixed in 10% formalin, embedded in paraffin, and serially sectioned. Standard hematoxylin and eosin (H&E) staining was used for visualization of cellular changes and examined by microscopy (Olympus ix71). Granuloma size was measured by computer-assisted morphometric analysis using DP-2BSW software (Olympus,Shinjuku, Tokyo, Japan). About 100 granulomas for each group were measured under a microscope. Granuloma size were measured by a blind analysis approach.

### Routine hematological analysis

Peripheral blood of mice was collected by EDTA-containing tubes. Blood samples were performed with the use of SYSMEX XS1000 automated analyzer.

### Biochemical assays

Serum was obtained by centrifugation at 800 *g* for 10 minutes. ALT and AST concentrations were determined using biochemical kits (Tellgen Life Technology, Shanghai, China).

### Elisa

Briefly, SEA was dissolved in coating buffer at a concentration of 80 μg/ml. Then, the plate was coated with SEA overnight at 4 °C. After washing, the plate was covered with 200 μl/well blocking solution at 37 °C for 1 hr. After washing, 100 μl of diluted serum was added to each well and incubated at 37 °C for 1 hr. After four washes, 100 μl HRP enzyme-labeled antibody solution diluted in PBS/Tween-20 was added and incubated at 37 °C for 1 hr. The plate was washed and the reaction was visualized by adding 100 μl TMB Substrate Reagent to each well for 10 min in the dark. The dilute sulfuric acid was added to terminate the reaction and the absorbance of each well was measured at 450 nm using an ELISA plate reader (Model ELX-800; BioTek).

### Statistics

Data were analyzed by SPSS 21.0 and statistical evaluation of the difference between means was assessed using one-way ANOVA and two-tailed Student’s t-tests. *P* < 0.05 was considered to be statistically significant.

## Result

### The expression of TLR3 increased significantly after *S. japonicum* infection

**Fig. 1:**
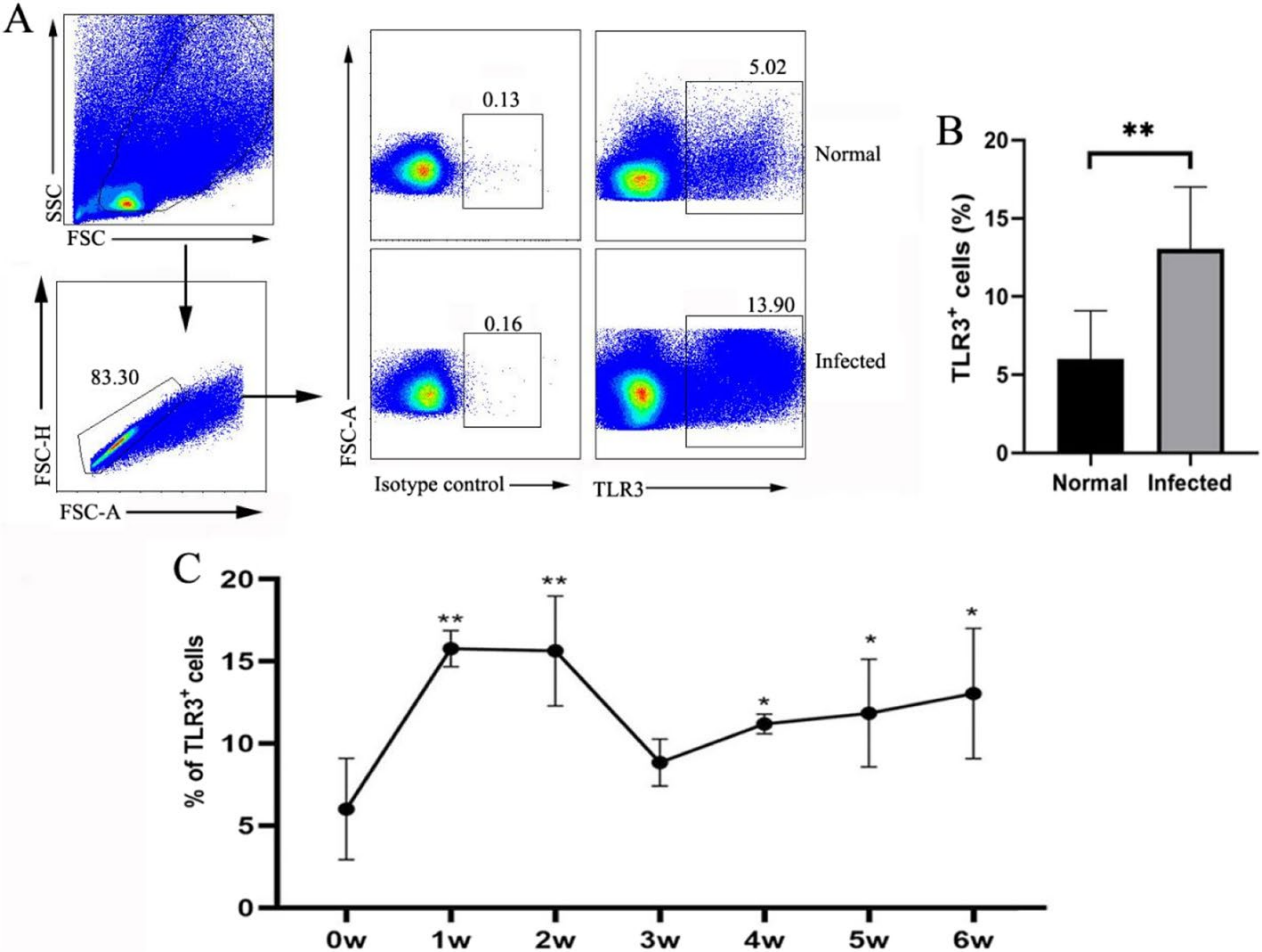
Evaluation of TLR3 expression during infection of *S. japonicum*. **A** Expression of TLR3 on hepatic cells was assessed by flow cytometry, gate strategy is shown. **B** Comparison between the normal and infected groups (infected with 40 ± 5 cercariae of *S. japonicum*), data representative of 6 to 7 independent results are shown. **C** C57BL/6 mice infected by *S. japonicum* were euthanized weekly from week 1 to week 6. The expression of TLR3 was dynamically detected by flow cytometry in the liver. (*P < 0.05, **P < 0.01, the error bars indicate SD)

To observe the expression of TLR3 during the course of *S. japonicum* infection, single cells were isolated from liver after infection and were examined by flow cytometry. Of the normal hepatic immune cells, the percentages of cells expressing TLR3 protein comprised 6.01 ± 3.08%. After infection, the TLR3 protein expression was significantly elevated compared with uninfected liver (**P < 0.01, Fig. 1A, B). The expression kinetics showed that the expression of TLR3 increased on week 1. After that, the percentages slightly decreased on week 3, followed by a second increase on week 4 that persists until week 6 (Fig. 1C). These results indicated that infection induced a significant increase of TLR3.

### Cellular distribution of TLR3 in the livers of infected mice

**Fig. 2:**
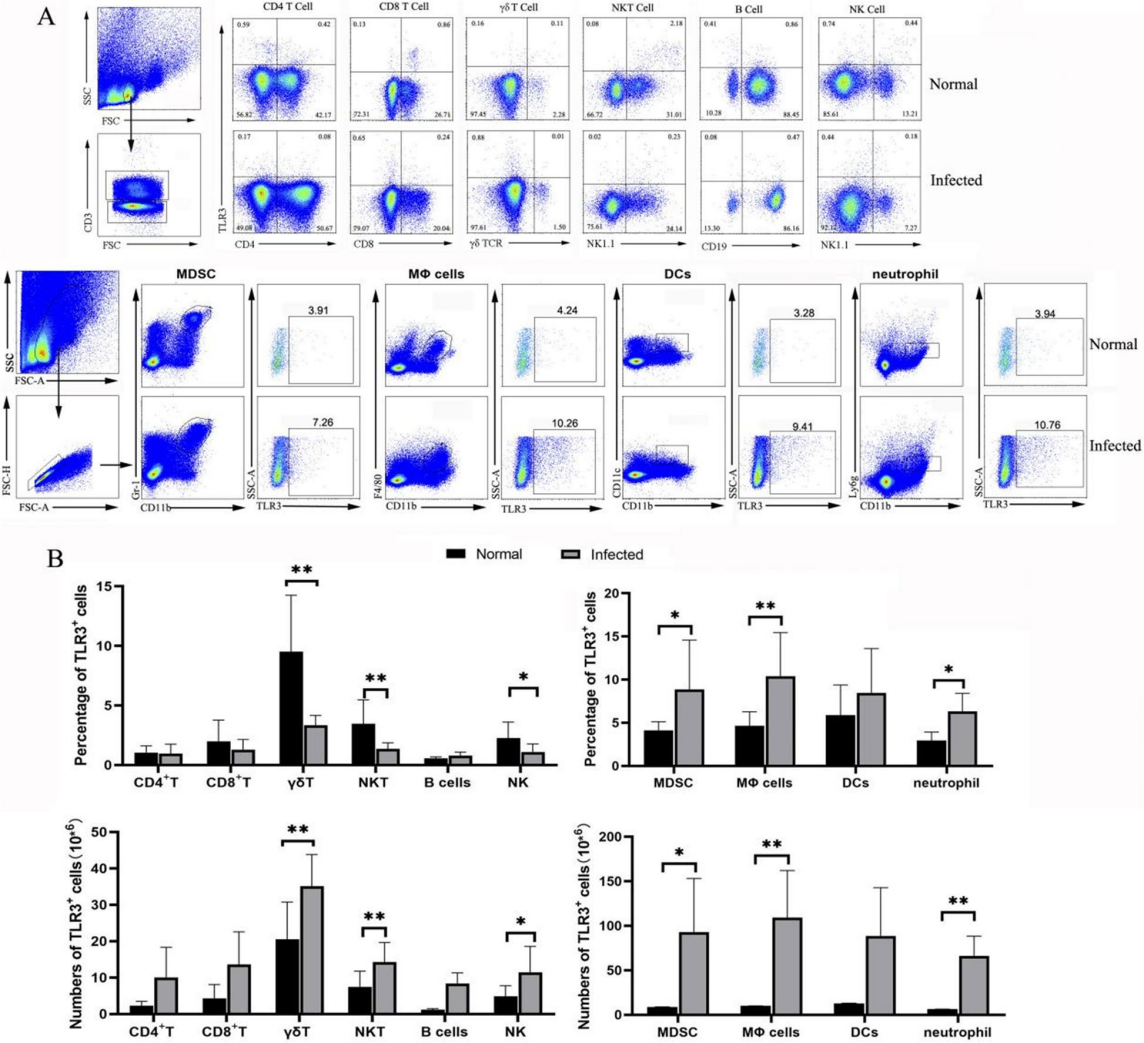
The distribution of TLR3 in different immune cells. WT mice were infected with *S. japonicum*, and livers were removed. Single-cell suspensions of liver were isolated from naive and infected mice. **A** Gate strategy for TLR3^+^ cells and CD4^+^T cells (CD4^+^CD3^+^), CD8^+^T cells (CD8^+^CD3^+^), γδT cells (γδTCR^+^CD3^+^), NKT cells (NK1.1^+^CD3^+^), B cells (CD19^+^CD3^−^), NK (NK1.1^−^CD3^+^) cells, MDSC (CD11b^+^Gr1^+^), macrophages (CD11b^+^F4/80^+^), DCs (CD11c^+^CD11b^+^) and neutrophils (CD11b^+^ Ly6g^+^) isolated from liver immune cells of WT and infected mice are shown. **B** The proportion and number of TLR3^+^ cells and CD4^+^T cells, CD8^+^ T cells, γδT cells, NKT cells, B cells NK cells, MDSC, macrophages, DCs and neutrophils from normal and infected mice are shown. Data were obtained from three independent experiments (*n* = 3–4) shown as the mean ± SD. *P < 0.05 and **P < 0.01

To evaluate TLR3 distribution in different immune cells, changes in TLR3 expression were determined after infection. Cells were isolated from normal and infected C57BL/6 mice livers. Firstly, cells were stained with different fluorophore conjugated Abs for CD3, CD4, CD8, γδTCR, NK1.1, CD19, CD11b, Gr1, F4/80, Ly6G and CD11c for FACS analysis. Lymphoid cells, including γδT cell, NK cell, and NKT cell populations, significantly expressed lower percent of TLR3 in the infected liver than that in the normal cell population (*P < 0.05, **P < 0.01, Fig. 2A, B). However, the percentage of TLR3^+^ cells in CD11b^+^Gr1^+^ (MDSC), CD11b^+^F4/80^+^ (macrophages), CD11b^+^CD11c^+^ (DCs) increased significantly in the infected mice (*P < 0.05, **P < 0.01). The absolute numbers of TLR3^+^ myeloid cells in the liver markedly increased, which was consistent with the above results. This indicated that TLR3 might mainly established its role through myeloid cells in the livers of *S. japonicum*-infected mice.

### TLR3 KO marginally lighten inflammation of liver

**Fig. 3:**
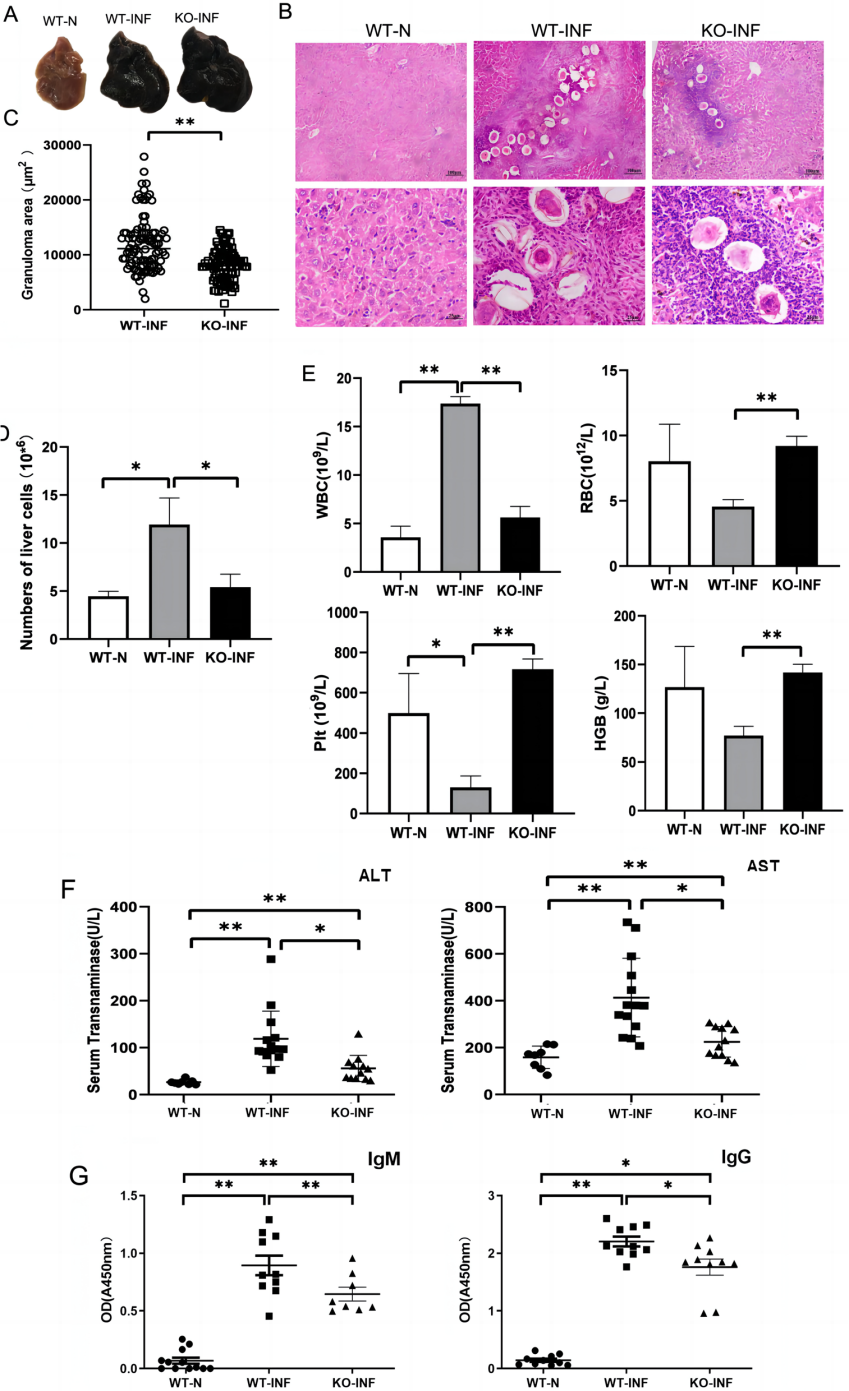
The pathological features of C57BL/6 mice during *S. japonicum* infection. WT and TLR3 KO mice were infected with 40 ± 5 cercariae of *S. japonicum*. Six weeks after the infection, the mice were euthanized. **A** The gross appearance of the three groups were observed. **B** Sections of the liver were staining by HE (original magnification × 100 for upper panels and × 400 for lower panels). The bar represents 100 μm in the upper panels and it represents 25 μm in the lower panels. **C** Granulomas size were calculated by computer-assisted morphometric analysis. About 100 granulomas per for each group were measured under a microscope and analysed using DP-2BSW software (Olympus, Shinjuku, Tokyo, Japan). **D** The absolute number of non-parenchymal liver cells from non-infected, infected WT and KO mice are shown. **E** Peripheral blood was collected and the numbers of white blood cells, red blood cells, platelet and the content of Hemoglobin were analyzed. **F** The levels of glutamic-pyruvic transaminase (ALT) and glutamic-oxalacetic transaminase (AST) in serum were detected. **G** The relative titers of anti-SEA IgG and anti-SEA IgM in the serum of non-infected and infected WT and KO mice are shown. (*P < 0.05, **P < 0.01)

To explore the role of TLR3 in mice infected with *S. japonicum*, wild-type (WT) and TLR3 knockout (KO) mice were infected with *S. japonicum* and then were sacrificed 6–7 weeks after infection. The appearance of normal liver was light red with a smooth surface following blood removal (Fig. 3A). In contrast, the WT infected liver was darker red with many small white spots on the surface, indicating severe inflammation and many pyogenic granulomas, while the differences between the appearance in the WT and KO infected livers were not obvious. Paraffin sections were made and stained with HE to observe the effects of infection on liver microstructure.

Results identified that there was obvious granuloma caused by egg deposition in liver of *Schistosoma japonicum*- infected mice (Fig. 3B). Few granulomas were found in KO infected livers compared with WT infected liver. WT infected livers developed larger granulomas, whereas these granulomas were significantly smaller in KO infected livers (*P* < 0.01, Fig. 3C). The number of non-parenchymal liver cells from WT infected mice were significantly higher than that in normal mice (*P < 0.05). Moreover, the number of non-parenchymal liver cells in infected TLR3 KO mice were markedly decreased compared to those in infected WT mice (*P < 0.05, Fig. 3D). As shown in Fig. 3E, the number of WBC were significantly reduced in TLR3 KO infected mice compared to WT infected mice (*P* < 0.01), but the levels of RBC, platelet and HGB were significantly increased in KO infected mice. There was no significant difference in WBC, RBC, platelet and HGB levels between normal WT mice and TLR3 KO infected mice. It is known that serum AST and ALT activities are sensitive indicators of hepatocytes’ injury. To observe the change of serum aminotransferase (ALT and AST) during the course of *S. japonicum* infection, blood was collected from both WT and TLR3 KO mice, and ALT and AST (Fig. 3F) and SEA specific IgM and IgG antibodies (Fig. 3G) were detected. The results indicated that the levels of ALT and AST increased significantly after infection (*P* < 0.01). They were significantly reduced in KO infected mice compared to WT infected mice (*P* < 0.05). The levels of SEA-specific IgM and IgG antibodies in WT and TLR3 KO infected mice were significantly higher than those in normal mice (*P < 0.05,**P < 0.01). Moreover, the levels of SEA specific IgM and IgG antibodies in the serum of infected TLR3 KO mice were markedly decreased compared to those in infected WT mice (*P < 0.05, **P < 0.01). These results suggested that TLR3 might accelerate the progression of *S. japonicum* infection-induced hepatitis.

### Phenotypic changes of lymphocytes in the liver of *S. japonicum*-infected TLR3 KO mice

**Fig. 4:**
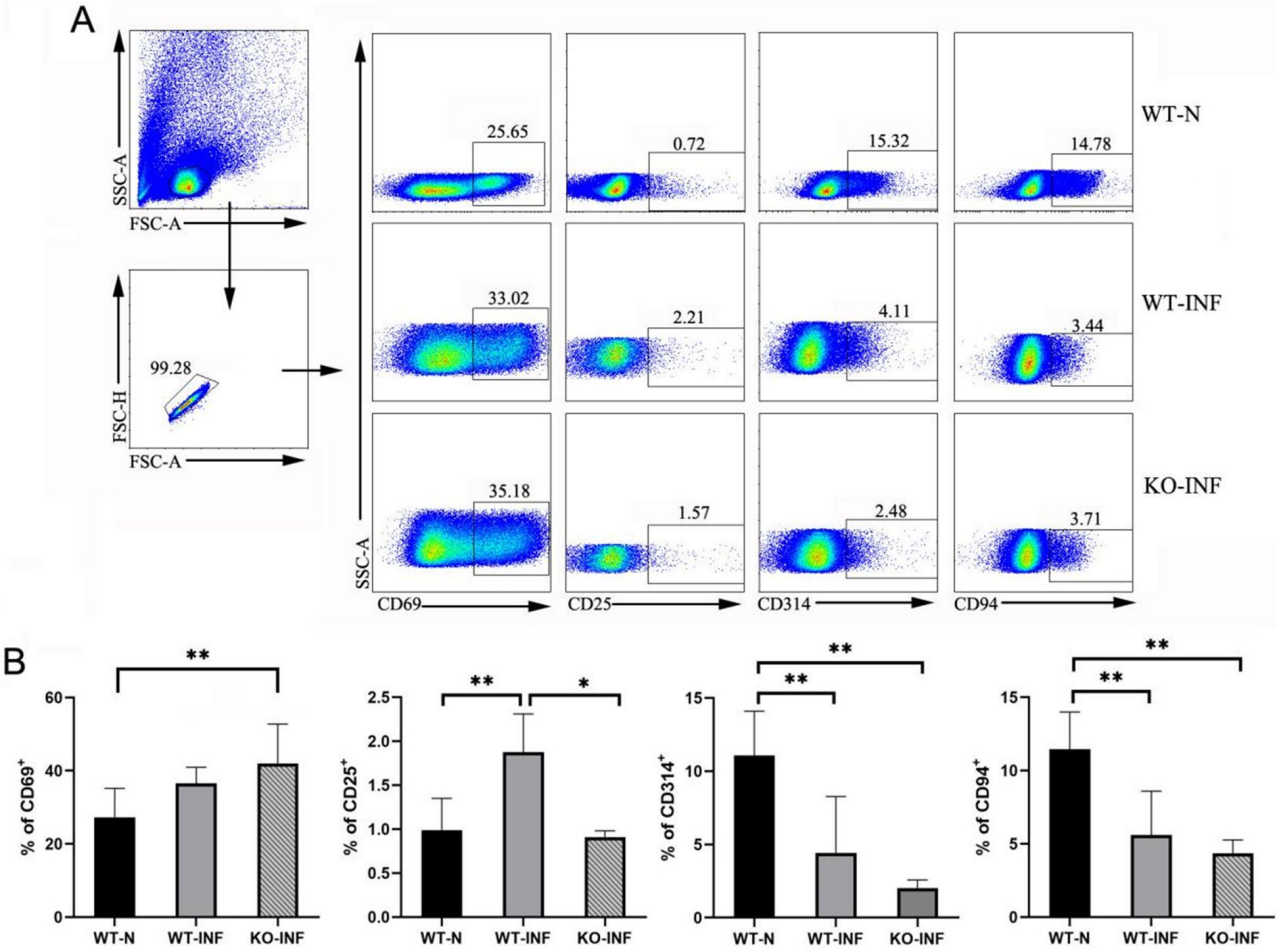
Phenotypic alterations in *S. japonicum*-infected TLR3 KO mice. WT and TLR3 KO mice were infected with 40 ± 5 cercariae of *S. japonicum*. Six weeks later, livers were collected. Single-cell suspensions were stained with monoclonal antibodies against mouse CD25, CD69, CD314, CD94 and analyzed by flow cytometry. **A** Gate strategy. **B** Average percentages were calculated from three independent experiments (*n* = 3), shown as the mean ± SD. (*P < 0.05, **P < 0.01)

To explore the characteristics of TLR3 during infection, the hepatic single cells from both WT and KO mice (6–7 weeks after infection) were prepared, and CD25, CD69, CD314 and CD94 were detected by FCM (Fig. 4). As shown in Fig. 4, it indicated that the percentage of CD69 was significantly higher in KO infected mice compared with uninfected control (*P* < 0.01), whereas there was no significant difference in CD69 levels between infected WT mice and infected KO mice. The percentage of CD25 from WT infected mice was significantly higher than of cells in both uninfected WT mice and KO infected mice (**P < 0.01,*P < 0.05). On the contrary, the proportions of CD94 and CD314 were decreased from WT infected mice liver, compared with uninfected WT mice (*P* < 0.01). Moreover, similar characteristic was observed in the proportions of CD314 and CD94 in the KO liver following *S. japonicum* challenge, which were decreased compared with uninfected WT mice. In brief, compared with infected WT mice, infected KO mice exhibited no significantly difference in the expression of CD69, CD314 and CD94 (*P* > 0.05), except for CD25 (*P* < 0.01).

## Discussion

The Toll-like receptor (TLR) pathway can trigger immune response to pathogen-associated molecular patterns (PAMPs) or endogenous molecules released in response to inflammation [28]. TLRs are type I integral membrane glycoproteins with an extracellular leucine-rich repeat domain [29], which are expressed on the cell surface or in intracellular organelle, such as lysosomes, endosomes and endoplasmic reticulum [30]. TLR3 is a receptor for double-stranded RNA (dsRNA) and is activated through the MyD88-independent pathway mediated by the TRIF adapter [31]. TLR3 has been identified as the major MyD88-independent PRR stimulated in the type-1 IFN responses to many different viral infections due to its intracellular localization. The academics pointed out that TLR3 has a major regulatory role during *Schistosoma mansoni* egg-driven Th2 responses in the lung [32] and it showed significant upregulation in the liver after *Schistosoma mansoni* infection [33]. The expression kinetics demonstrated the existence of *S. japonicum* infection-induced TLR3 expression in C57BL/6 mouse livers at early time points following infection. Moreover, this trend persists throughout the course of infection, although slightly decreased on week 3. Our findings demonstrated that TLR3 might participate of the immune during *S. japonicum* infection.

TLR3 is expressed on a variety of immune cells, such as T cells, B cells and macrophages, dendritic cells (DCs), but also in various other cell types including pancreatic epithelial cells [34, 35]. TLR3 signals also promote the class-switching of B cells via DC and CD4^+^ T cells activation to elevate IgA production [36]. Current immunological dogma proposes a role for TLR3 signaling in maintaining the integrity of epithelial barrier function during genital tract Chlamydia infection [37]. This may be related to the fact that the role of TLR3 in different diseases and organs was different. In this study, the expression of TLR3 was examined on different cell types from liver from normal and *S. japonicum*-infected mice. Macrophages, MDSCs, and neutrophils are myeloid cells that express many kinds of TLRs, including TLR3, and regulate the adaptive immune response [38, 39]. Consistent with previous reports, the percentage of TLR3^+^ MDSCs, TLR3^+^macrophages and TLR3^+^neutrophils increased significantly in the livers of *S. japonicum-*infected mice. These results implied that TLR3 might regulate the *S. japonicum* infection induced immune response mainly through myeloid cells,like macrophages, MDSCs, and neutrophils. The expression of TLR3 on NK cells analysis in the infected liver was lower compared to normal group It differed with a previous study [27], in which it is up-regulated in splenic NK cells from *S. japonicum*-infected mouse. One potential reason for this discrepancy might be tissue specificity.

It is believed that the immune responses of humans to schistosome eggs and granuloma are the major causes of pathology in schistosomiasis [40]. The granulomas damage hepatocytes and the normal histological structure, consequently induce portal hypertension. TLR3 is involved in the host response to viral infection [41], and has a possible role during *Plasmodium* infection through the initiation of complex circuits and signals of the immune response [42]. In this study, gross examination of livers excised indicated that infection caused inflammatory cell infiltration that was no significant difference between WT infected and TLR3 KO infected mice. Hepatic granulomatous inflammation was significantly reduced in TLR3 KO mice as measured by granuloma cross-sectional area. We demonstrated that TLR3 deficiency led to reduced numbers of peripheral blood WBC and restored the peripheral blood RBC numbers, platelet and HGB content of infected mice, which suppressed the development of schistosomiasis. Aspartate aminotransferase (AST) and alanine aminotransferase (ALT) mainly exist in the cytoplasm of hepatocytes and are released into the blood when they are damaged [43], which are important diagnostic indexes of liver fibrosis [44, 45]. In our study, it was shown that absence of TLR3 reduces the levels of AST and ALT during infection. Moreover, the serum titles of SEA-specific IgMs and IgGs decreased significantly in TLR3 KO mice compared with WT mice during infection. These results demonstrated a positive relationship between TLR3 and the severity of liver pathogenesis, and consistent with previous reports [27] implicating TLR3 in the granulomatous inflammatory reaction during *S. japonicum* infection.

CD69, CD25, CD314 and CD94 are function- and activation- associated molecules which were expressed on the surface of many immune cells, such as natural killer, B cells, (NK) cells, monocytes, neutrophils and also eosinophil [6, 46]. CD69 is one of the early activation in the cells, the higher level of it in TLR3 KO mice may mainly reflect that the absence of TLR3 signaling trigger mechanisms early activation status during infection. CD25 is proposed to be a phenotypical marker of regulatory T cells and also expressed on both activated cells and some regulatory cells [6]. Therefore, the upregulation of CD25 we observed in infected WT mice compared with infected TLR3 KO mice might be either due to activation or increased receptor expression based on the regulatory background of TLR3 during *S. japonicum* infection. The CD25-relatived activated function might be in close contact with TLR3 expression. It indicated that TLR3 is involved in the mechanism of activating in the model of *S. japonicum.*

In summary, TLR3 expression showed significant up-regulation in the liver of infected mice in contrast to the normal group. It suggested that TLR3 might be involved in regulating the immune response in the course of *S. japonicum* infection in C57BL/6 mice. Loss of TLR3 partially alleviate the liver damage during infection.

## Data Availability

The datasets in the current study are included in the published article or available from the corresponding author on reasonable request.
